# A Liquid Chromatography-High Resolution Mass Spectrometry (LC-HRMS) Method for the Determination of Free Hydroxy Fatty Acids in Cow and Goat Milk

**DOI:** 10.3390/molecules25173947

**Published:** 2020-08-29

**Authors:** Maroula G. Kokotou, Christiana Mantzourani, Asimina Bourboula, Olga G. Mountanea, George Kokotos

**Affiliations:** Department of Chemistry, National and Kapodistrian University of Athens, 15771 Athens, Greece; mkokotou@chem.uoa.gr (M.G.K.); chrmantz@chem.uoa.gr (C.M.); minabour@chem.uoa.gr (A.B.); olgamount@chem.uoa.gr (O.G.M.)

**Keywords:** determination, hydroxy fatty acids, high resolution mass spectrometry, liquid chromatography, milk

## Abstract

A liquid chromatography–high resolution mass spectrometry (LC-HRMS) method for the direct determination of various saturated hydroxy fatty acids (HFAs) in milk was developed for the first time. The method involves mild sample preparation conditions, avoids time-consuming derivatization procedures, and permits the simultaneous determination of 19 free HFAs in a single 10-min run. This method was validated and applied in 17 cow milk and 12 goat milk samples. This work revealed the existence of various previously unrecognized hydroxylated positional isomers of palmitic acid and stearic acid in both cow and goat milk, expanding our knowledge on the lipidome of milk. The most abundant free HFAs in cow milk were proven to be 7-hydroxystearic acid (7HSA) and 10-hydroxystearic acid (10HSA) (mean content values of 175.1 ± 3.4 µg/mL and 72.4 ± 6.1 µg/mL in fresh milk, respectively). The contents of 7HSA in cow milk seem to be substantially higher than those in goat milk.

## 1. Introduction

Lipids are one of the most important classes of milk components [1,2]. Apart from their role as a source of energy, they contribute to the sensory and physical properties of milk. The majority of lipids in dairy fat are triacylglycerols [3], while small amounts of free fatty acids (FFAs) are also present as a result of the enzymatic hydrolysis of triacylglycerols by lipoprotein lipase and other lipolytic enzymes [4]. Among the numerous fatty acids (FAs) found in milk, long-chain saturated fatty acids (SFAs) are the predominant class [5,6]. However, common unsaturated FAs (mainly mono-unsaturated) as well as short-chain FAs, branched, and odd-chain FAs, trans FAs, and conjugated linoleic acid are also present [7,8].

Various FAs present in milk fat are linked either in a positive or a negative way with human health [9]. For decades, nutritional guidelines have suggested a reduction in the intake of milk SFAs, because these fatty acids have been linked with pathologies such as metabolic syndrome and cardiovascular diseases. This notion is currently in question, and recent scientific studies do not seem to justify such recommendations in a healthy population [10,11,12]. Most recently, Astrup and colleagues argued that the health effects vary for different types of SFAs and that the composition of the food source is crucially important [13]. For example, higher levels of odd-chain SFAs C15:0 and C17:0 are associated with a lower risk of type 2 diabetes (T2D), as shown by a recent large meta-analysis which pooled the findings from 16 prospective cohort studies [14].

The analytical methods, for the determination of free fatty acids (FFAs) in milk have been recently reviewed [6,15]. Gas chromatography-mass spectrometry (GC-MS) analysis is the most popular technique for the analysis of FFAs, after their conversion into their corresponding methyl esters (FAMEs). A high performance liquid chromatography-mass spectrometry (HPLC-MS) method has been reported for the determination of FFAs in food samples, including milk, after derivatization with 2-hydrazinoquinoline [16], while most recently we employed a LC-High Resolution MS (LC-HRMS) method for the quantification of FFAs in milk [17].

The presence of saturated hydroxy fatty acids (SHFAs) in foods has attracted less attention so far. Jenske and Vetter have described the analysis of 2- and 3-hydroxy FAs (2HFAs, 3HFAs) in food samples, and they have determined the concentrations of several 2HFAs and 3HFAs in milk and other dairy products by gas chromatography with electron-capture negative ion mass spectrometry [18,19]. Only one report refers to the existence in milk of two different SHFAs possessing the hydroxyl at higher positions of the fatty chain—namely, 10-hydroxystearic acid and 8-hydroxypalmitic acid [20].

Within our project dedicated to the synthesis and study of bioactive lipids, we have a special interest in SHFAs, since such compounds are the key components of a recently identified novel class of anti-inflammatory and anti-diabetic lipids, called the fatty acid esters of hydroxy fatty acids (FAHFAs) [21,22,23]. However, SHFAs exhibit biological roles and actions by their own right. For example, 9-hydroxystearic acid (9HSA) has been shown to inhibit cell growth in human colon cancer, targeting histone deacetylase−1 [24], and to mediate the switch from proliferation to differentiation in retinal progenitor cells [25]. We have recently developed methods for the synthesis of various SHFAs, including both 9-hydroxy and 3-hydroxy FAs [26,27].

The aim of the present work was the development of a LC-HRMS method for the straightforward and rapid determination of various free SHFAs in milk samples. Such a method may unravel the existence of potential bioactive ingredients in milk that may play a role in human health. We describe herein a method allowing the simultaneous determination of 19 SHFAs in a single 10-min run, avoiding any derivatization step and employing a simple and mild protocol for sample preparation.

## 2. Results and Discussion

### 2.1. LC-ESI-MS Data

Nineteen SHFAs were used in the present study. Eight of them were regio-isomers of hydroxy palmitic acid [2-hydroxypalmitic acid (2HPA), 3-hydroxypalmitic acid (3HPA), 7-hydroxypalmitic acid (7HPA), 8-hydroxypalmitic acid (8HPA), 9-hydroxypalmitic acid (9HPA), 10-hydroxypalmitic acid (10HPA), 11-hydroxypalmitic acid (11HPA), 16-hydroxypalmitic acid (16HPA)], and seven were regio-isomers of stearic acid [2-hydroxystearic acid (2HSA), 3-hydroxystearic acid (3HSA), 7-hydroxystearic acid (7HSA), 8-hydroxystearic acid (8HSA), 9-hydroxystearic acid (9HSA), 10-hydroxystearic acid (10HSA), 12-hydroxystearic acid (12HSA)]. In addition, four 3-hydroxy FAs [3-hydroxycapric acid (3HCA), 3-hydroxylauric acid (3HLA), 3-hydroxymyristic acid (3HMA), 3-hydroxypentadecanoic acid (3HPDA)] were included in the set of compounds tested. The high-resolution mass spectra of these HFAs were recorded in electrospray ionization (ESI) negative mode. The structures of the SHFAs used along with the exact masses of the deprotonated molecules and the retention times observed for each one of them in the chromatographic method are presented in Table 1. The MS/MS spectra of all the 19 analytes in negative ESI mode are also presented in the Supplementary Materials (Figure S1).

**Figure 1:**
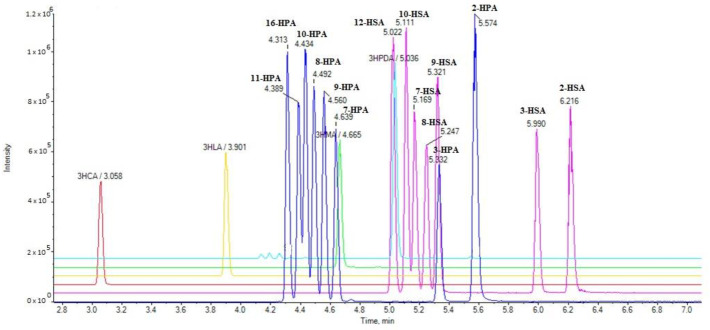
Extracted ion chromatograms (EICs) of hydroxy fatty acids in a standard solution (1 µg/mL).

We have developed a rapid LC-HRMS method allowing the simultaneous determination of a variety of SHFAs in milk in a 10-min run. The extracted ion chromatograms (EICs) of the analytes in a standard solution (1 µg/mL) are presented in Figure 1.

### 2.2. Method Validation

Good linearity values ranging from 0.990 to 0.998 were observed for all the analytes. The limits of detection (LOD) and quantification (LOQ) are summarized in Table 2. The limits of detection varied from 0.1 to 0.9 ng/mL, while the limits of quantification ranged from 0.4 to 2.6 ng/mL.

A simple sample preparation procedure was followed, involving the addition of methanol for protein precipitation. After centrifugation, the supernatant was used for the analysis. For the verification of the accuracy and precision, the guidelines of the EU Commission decision 202/657/EC were followed. The milk samples were spiked at three different concentration levels with three replicates for each fortification level. Satisfactory recoveries, ranging from 80.8 to 109.4 for the low spike level, from 81.4 to 109.1 for the medium spike level, and from 80.8 to 100.5 for the high spike level (Table 3), indicate the accuracy of the proposed method. The matrix factor was calculated as the ratio of the peak response in the presence of a matrix to the peak response in the pure solvents. A matrix factor value <1 suggests signal suppression in the samples, while a matrix factor value >1 denotes signal enhancement (Table 3). The precision was investigated by means of the relative standard deviation (%RSD). The %RSD values that were obtained for the intra-day (RSDr) and inter-day (RSD_R_) variations ranged from 0.54 to 11.16 and from 0.71 to 13.25, respectively, depending on the HFAs (Table 3).

### 2.3. Analysis of Samples

**Figure 2:**
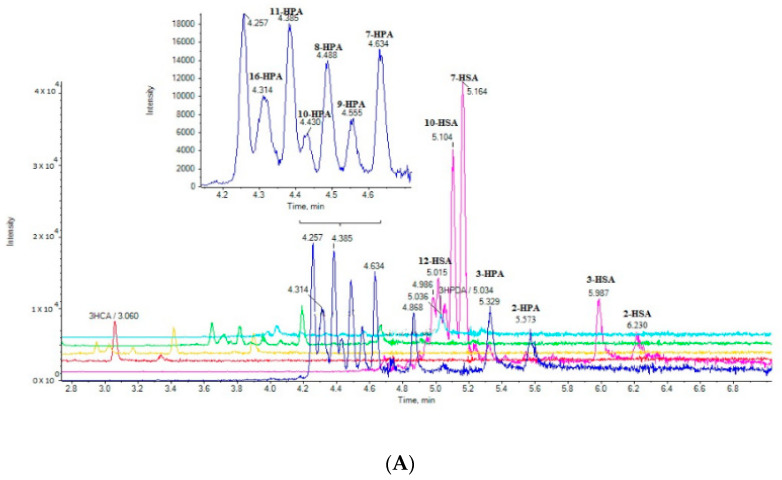

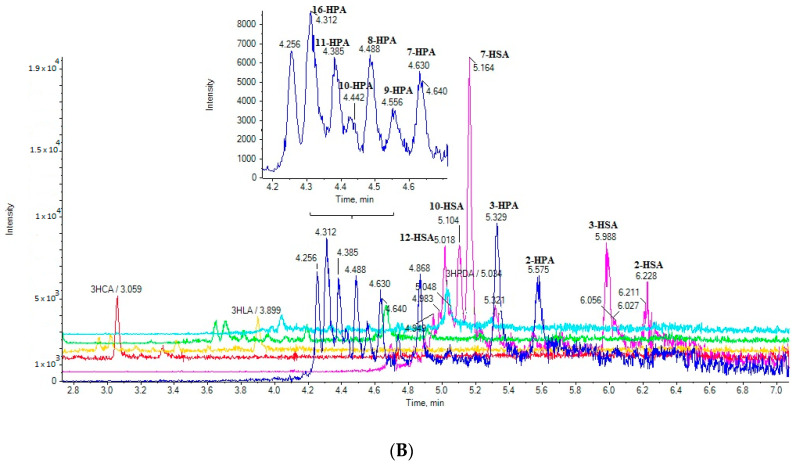
EICs of the analytes in a cow milk sample (**A**) and in a goat milk sample (**B**).

Seventeen cow milk samples and 12 goat milk samples were purchased from the local market and analyzed. The extracted ion chromatograms (EICs) of a cow milk sample (A) and a goat milk (B) sample are shown in Figure 2. The simultaneous determination of 19 SHFAs was achieved in a single 10-min run. The contents of the 19 analytes in milk samples (in triplicate) are summarized in Table 4, and they are expressed as the µg of HFA per mL of milk. The integration of the peak areas was performed manually using MultiQuant 3.0.2. As Hill et al. discussed [28], in particular cases a manual baseline may be employed for the quantification of the peak areas. The same integration parameters were used in all cases.

A variety of SHFAs were found to be present in both cow and goat milk (Table 4), in addition to 2HFAs and 3HFAs, which have been previously reported as milk fat minor components [18,19]. In cow milk, 11HPA was estimated as the most abundant (42.9 ± 4.7 µg/mL) among HPAs, followed by 16HPA, 3HPA, 8HPA, 2HPA, 10HPA, and 9HPA at levels varying from 37.2 ± 5.1 to 14.7± 7.1 µg/mL. 7HPA was absent from seven cow milk samples. The most abundant HFAs in cow milk were proven to be 7HSA and 10HSA. Their contents were estimated at mean values of 175.1 ± 3.4 µg/mL and 72.4 ± 6.1 µg/mL, respectively. 3HSA, 2HSA, and 9HSA were found to be present at levels varying from 33.8 ± 6.6 to 20.1 ± 4.7 µg/mL, while 8HSA was absent from all the cow milk samples and 12HSA absent from seven samples.

In goat milk, 3HPA was estimated as the most abundant (41.5 ± 2.7 µg/mL) among the HPAs, followed by 16HPA, 8HPA, 2HPA, 11HPA, and 9HPA in decreasing order, while 10HPA was estimated approximately at 5.8 ± 1.1 µg/mL, and 7HPA was absent from 10 samples. As in cow milk, 7HSA and 10HSA were found again to be the most abundant (38.2 ± 8.2 µg/mL and 39.1 ± 7.2 µg/mL, respectively), however at levels not as high as in cow milk. 8HSA was absent from all the samples.

The results highlight a remarkable difference in the contents of 7HSA and 10HSA in cow milk and goat milk. The contents of 7HSA in cow milk (minimum value 38.3 µg/mL, maximum value 378.8 µg/mL, mean value 175.1 µg/mL) seem to be substantially higher than those in goat milk (minimum value 21.5 µg/mL, maximum value 48.0 µg/mL, mean value 38.2 µg/mL). Thus, 7HSA might be a potential marker to discriminate cow milk from goat milk. The contents of 10HSA in cow milk (minimum value 31.2 µg/mL, maximum value 250.1 µg/mL, mean value 71.4 µg/mL), although not presenting such a marked difference, are again higher than those in goat milk (minimum value 18.0 µg/mL, maximum value 75.4 µg/mL, mean value 39.1 µg/mL).

Regarding the 3HFAs studied in this work (3HCA, 3HLA, 3HMA, 3HPDA, 3HPA, and 3HSA), all of them were found to be present in goat milk, while 3HCA, 3HLA, 3HMA, and 3HPDA were absent from 8, 7, 8, and 13 milk samples, respectively (Table 4).

**Figure 3:**
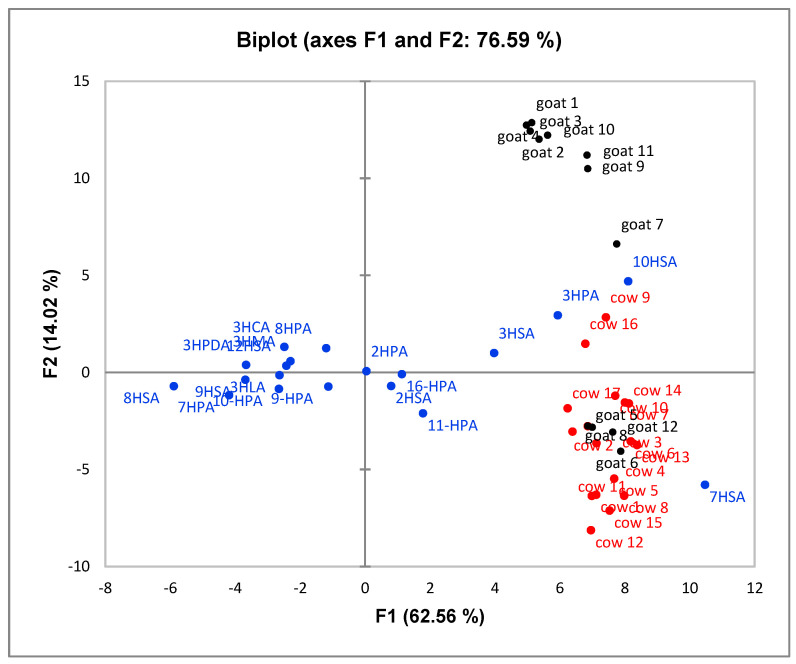
Principal component analysis (PCA) biplot graph of HFAs from cow and goat milk. Active variables (cow red, goat black), active observations (blue).

Principal Component Analysis (PCA) was performed to explore the relationship between the samples and the data. The results indicated that component PC1 (62.56%) and component PC2 (14.02%) were accountable of the total variance and presented Eigen values 18.14 for PC1 and 4.07 for PC2. The Scree plot is depicted in Supplementary Materials (Figure S2). The biplot graph is illustrated in Figure 3. Although a perfect discrimination of both groups of milk samples was not accomplished, a general trend was observed with the majority of cow milk samples to be located at the lower right part of the plot, while the majority of goat milk samples tend to be located at the upper right part of the plot. 7HSA and 11HPA contents (lower right part in the graph) seem to be well correlated with cow milk samples, while 10HSA and 3HPA contents (upper right part in the graph) with goat milk samples.

The method developed herein allowed us to study for the first time the existence of various hydroxylated positional isomers of palmitic acid and stearic acid in milk and to expand our knowledge on the lipidome of milk. Having on hand a variety of HFAs that previously synthesized by us [26,27], we were able to demonstrate that two families of previously unrecognized HFAs are present in milk, HPAs and HSAs, each one consisting of various members carrying the hydroxyl functionality at different chain positions.

In addition, the simultaneous determination of six free 3HFAs (3HCA, 3HLA, 3HMA, 3HPDA, 3HPA, 3HSA) and two 2HFAs (2HPA and 2HSA) was achieved, following a simple sample preparation protocol. The previous report by Jenske and Vetter referred to the analysis of total 3HFAs and 2HFAs following a tedious sample preparation procedure [19]. The extracted saponifiable lipids were converted into methyl esters, and the resulting FAMEs were separated by chromatography into hydroxy-FAMEs (OH-FAMEs) and non-OH-FAMEs. Then, the OH-FAMEs were converted to pentafluorobenzoyl derivatives and analyzed by gas chromatography with electron capture negative-ion mass spectrometry (GC/ECNI-MS) in the selected ion monitoring (SIM) mode [19] Thus, the present method has clearly the advantage of rapidness and simplicity.

## 3. Materials and Methods

### 3.1. Chemicals and Reagents

All the solvents used were of LC-MS analytical grade. Acetonitrile was purchased from Carlo Erba (Val De Reuil, France), isopropanol and methanol from Fisher Scientific (Laughborough, UK), and formic acid 98–100% from Chem-Lab (Zedelgem, Belgium). 2HPA and 2HSA were commercially available from Cayman Chemical (Michigan, USA), and 16HPA from Sigma-Aldrich (Darmstadt, Germany). 3HCA, 3HLA, 3HMA, 3HPDA, 3HPA, and 3HSA were synthesized as previously described, and their analytical data were in accordance with the literature [27]. 9HPA, 9HSA, 11HPA, and 12HSA were synthesized as previously described, and their analytical data were in accordance with the literature [26]. 7HPA, 8HPA, 10HPA, 7HSA, 8HSA and 10HSA were synthesized following the general method previously described by us [26] and their details will be presented elsewhere.

### 3.2. Stock and Working Solutions

Stock solutions of the standard compounds (1000 mg/L in methanol) were prepared and stored at 4 °C. Working standard solutions (500 ng/mL) were prepared daily by appropriate dilution.

### 3.3. Instrumentation

Chromatography was performed on a Halo C18 column (2.7 μm, 90 Å, 0.5 × 50 mm) from Eksigent, using a micro-LC Eksigent (Eksigent, Darmstadt, Germany) equipped with an autosampler set at 5 °C and a thermostated column compartment. Separation was performed with a gradient over the course of 10 min at a flow rate of 55 µL/min, using a mobile phase system consisting of solvent A: acetonitrile/0.01% formic acid/isopropanol 80/20 v/v and solvent B: H_2_O/0.01% formic acid. The gradient elution program was as follows: 0–0.5 min, 5% B; 0.5–8.0 min, gradually increasing to 98% B; 8.0–8.5 min, 98% B, followed by a 1.5 min equilibration step to the initial conditions prior to the next injection. The injection volume was set at 5 µL.

An ABSciex Triple TOF 4600 (ABSciex, Darmstadt, Germany) was used to perform the HRMS measurements and all the experiments were carried out by ESI in negative mode. The data acquisition method consisted of a TOF-MS full scan *m/z* 50–850 and several information-dependent acquisition (IDA)-TOF-MS/MS product ion scans, using a 40 V collision energy (CE), with a 15 V collision energy spread (CES) used for each candidate ion in each data acquisition cycle (1091). The MS resolution working conditions were: ion energy 1 (IE1) −2.3, vertical steering (VS1) −0.65, horizontal steering (HST) 1.15, and vertical steering 2 (VS2) 0.00. MultiQuant 3.0.2 and PeakView 2.1 (ABSciex, Darmstadt, Germany) were employed for the data acquisition. EICs were obtained creating the base peak chromatograms for masses that achieve a 0.01 Da mass accuracy width. The relative tolerance of the retention time was set within a margin of ± 2.5%.

### 3.4. Sample Preparation

Μethanol (4 mL) was added to the milk sample (1 mL) in a screw cap glass centrifuge tube. After stirring for about 30 s, the sample was centrifuged at 4000× *g* for 10 min. The supernatant (500 µL) was then mixed with 500 µL of water in a vial, and this mixture was used for the LC-MS/MS analysis.

### 3.5. Method Validation

Solutions from 5–800 ng/mL of 2HPA, 3HPA, 7HPA, 8HPA, 9HPA, 10HPA, 11HPA, 16HPA, 2HSA, 3HSA, 7HSA, 8HSA, 9HSA, 10HSA, 12HSA, 3HCA, 3HLA, 3HMA, and 3HPDA (3 replicates; 13 levels (5, 10, 30, 50, 80, 100, 200, 300, 400, 500, 600, 700, 800 ng/mL); *n* = 3 × 13) were used to assess the linearity and the limits of detection (LOD) and quantification (LOQ). The LOD and LOQ were calculated using the signal-to-noise method. A signal-to-noise ratio (S/N) of three is generally accepted for estimating the LOD, and a signal-to-noise ratio of 10 is used for estimating the LOQ. This method is commonly applied to analytical methods that exhibit baseline noise.

Cow milk samples were spiked at three different concentration levels to estimate the recovery and the intra-day and inter-day variations. The recovery was used for the quantification of the selected compounds in milk.

### 3.6. Milk Samples

Twenty-nine brand products of fresh (pasteurized) whole milk were collected from the local market in Athens, Greece. Seventeen of them were cow milk products, and 12 of them were goat milk products.

### 3.7. Statistical Analysis

Level of significance was estimated using Excel t-Test: two-sample assuming unequal variances. Principal Component Analysis (PCA) was performed using XLSTAT (2018).

## 4. Conclusions

In conclusion, we demonstrate herein for the first time a method for the determination of various free HFAs, including palmitic and stearic acids carrying the hydroxyl group at different positions, in cow and goat milk. Our LC/HRMS method involves mild sample preparation conditions, avoids time-consuming extraction pre-separation or derivatization procedures, and permits the quantification of 19 free HFAs in a single 10-min run. Using this rapid and robust method, various previously unrecognized hydroxylated palmitic and stearic acids were identified both in cow and in goat milk. The most abundant HFAs in cow milk were proven to be 7HSA and 10HSA (mean content values of 175.1 ± 3.4 µg/mL and 72.4 ± 6.1 µg/mL in fresh milk, respectively). The contents of 7HSA in cow milk seem to be substantially higher than those in goat milk, and thus 7HSA might be a potential marker to discriminate cow milk from goat milk. Given that HFAs are an emerging class of bioactive lipids, the present method is of particular interest in identifying novel lipids that may play a role in human health.

## Figures and Tables

**Table 1:** Hydroxy fatty acid standards used in the chromatographic method.

Compound	Abbreviation	Structure	Theoretical Mass [M−H]^-^	Retention Time (min)
2-Hydroxypalmitic acid	2HPA	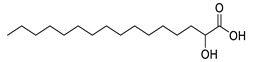	271.2279	5.57
3-Hydroxypalmitic acid	3HPA	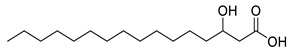	271.2279	5.33
7-Hydroxypalmitic acid	7HPA	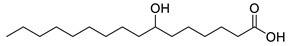	271.2279	4.64
8-Hydroxypalmitic acid	8HPA	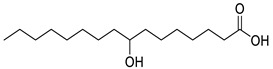	271.2279	4.49
9-Hydroxypalmitic acid	9HPA	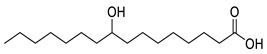	271.2279	4.56
10-Hydroxypalmitic acid	10HPA	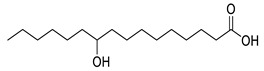	271.2279	4.43
11-Hydroxypalmitic acid	11HPA	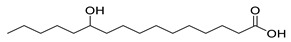	271.2279	4.39
16-Hydroxypalmitic acid	16HPA	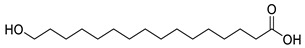	271.2279	4.31
2-Hydroxystearic acid	2HSA	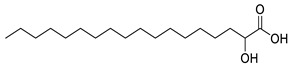	299.2592	6.22
3-Hydroxystearic acid	3HSA	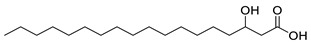	299.2592	5.99
7-Hydroxystearic acid	7HSA	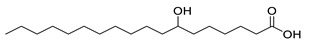	299.2592	5.17
8-Hydroxystearic acid	8HSA	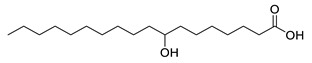	299.2592	5.25
9-Hydroxystearic acid	9HSA	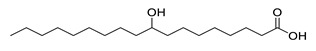	299.2592	5.32
10-Hydroxystearic acid	10HSA	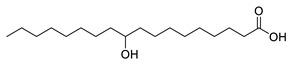	299.2592	5.11
12-Hydroxystearic acid	12HSA	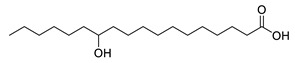	299.2592	5.02
3-Hydroxycapric acid	3HCA	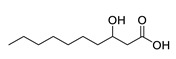	187.1340	3.06
3-Hydroxylauric acid	3HLA	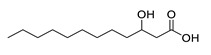	215.1653	3.90
3-Hydroxymyristic acid	3HMA	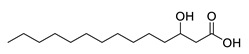	243.1966	4.67
3-Hydroxypentadecanoic acid	3PDA	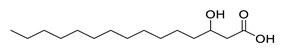	257.2122	5.04

**Table 2:** Limits of detection (LOD) and quantification (LOQ).

Analyte	LOD (ng/mL)	LOQ (ng/mL)
2HPA	0.3	0.8
3HPA	0.5	1.4
7HPA	0.8	2.4
8HPA	0.5	1.2
9HPA	0.6	1.7
10HPA	0.5	1.2
11HPA	0.5	1.6
16HPA	0.9	2.6
2HSA	0.1	0.4
3HSA	0.3	0.9
7HSA	0.4	1.1
8HSA	0.5	1.0
9HSA	0.6	1.8
10HSA	0.4	1.1
12HSA	0.5	1.4
3HCA	0.3	1.0
3HLA	0.5	1.4
3HMA	0.6	1.7
3HPDA	0.6	1.9

**Table 3:** Accuracy (recovery %), matrix factor (MF), and precision data (RSD %) in the spiked milk samples.

Analyte	Spike Level (ng/mL)	Recovery (%)	Matrix Factor (MF)	RSD_r_ (%)	RSD_R_ (%)
2HPA	10 300 500	80.8 101.4 100.5	0.7 0.9 1.0	8.53 10.50 4.06	2.11 8.79 5.12
3HPA	10 300 500	81.2 92.5 96.9	0.7 0.8 1.2	3.96 7.65 7.20	2.11 11.64 9.10
7HPA	10 300 500	94.9 95.5 96.1	1.0 0.9 1.2	4.51 3.78 8.78	4.31 7.65 9.13
8HPA	10 300 500	83.2 107.9 105.1	0.9 1.0 1.1	4.60 4.09 3.21	7.93 5.94 7.63
9HPA	10 300 500	103.1 109.1 100.00	1.1 1.0 1.3	0.54 5.51 4.96	0.76 8.88 6.21
10HPA	10 300 500	95.5 94.9 98.1	0.9 0.7 1.1	2.69 4.36 2.54	6.56 10.9 5.61
11HPA	10 300 500	109.3 90.8 95.3	0.9 0.8 1.2	1.42 5.12 1.11	6.29 6.36 4.32
16HPA	10 300 500	100.3 96.3 90.8	1.0 0.9 1.2	0.49 5.63 7.94	1.94 7.14 9.21
2HSA	10 300 500	92.6 88.0 92.5	1.1 0.5 1.3	0.71 9.74 11.10	3.79 13.25 12.73
3HSA	10 300 500	80.6 81.4 96.4	0.7 0.7 1.4	0.71 6.60 11.16	6.01 8.53 13.02
7HSA	10 300 500	104.9 103.0 88.1	1.2 0.9 0.5	5.14 2.41 9.06	7.03 11.2 12.1
8HSA	10 300 500	95.4 84.2 94.3	0.9 0.8 1.1	9.38 4.56 3.22	11.55 12.2 8.79
9HSA	10 300 500	92.6 85.7 90.9	0.9 0.8 1.4	0.27 9.74 9.06	0.71 13.25 10.41
10HSA	10 300 500	82.8 85.3 92.5	0.9 0.7 0.5	2.41 7.55 6.18	3.37 9.47 9.25
12HSA	10 300 500	109.4 100.9 101.6	1.1 1.0 1.2	3.37 6.18 3.42	10.12 7.01 5.21
3HCA	10 300 500	105.9 84.3 91.4	1.1 0.8 1.3	1.36 4.37 5.49	3.21 8.62 7.24
3HLA	10 300 500	89.4 89.7 90.3	0.9 0.8 1.3	4.16 8.62 3.51	4.78 6.07 4.21
3HMA	10 300 500	90.0 88.1 96.3	0.9 0.8 1.3	1.03 6.99 5.49	1.54 10.92 10.11
3HPDA	10 300 500	82.4 99.2 96.2	0.7 1.0 1.4	1.54 4.48 0.99	4.16 10.92 5.21

RSD_r_: intra-day relative standard deviation; RSD_R_: inter-day relative standard deviation.

**Table 4:** Contents of free hydroxy fatty acids in cow milk and goat milk samples (μg/mL fresh milk).

	Cow Milk (*n* = 17), Triplicates					Goat Milk (*n* = 12), Triplicates		
Hydroxy Fatty Acid	Minimum Value (μg/mL)	Maximum Value (μg/mL)	Mean Value ± SD (μg/mL)	Level of Significance	Minimum Value (μg/mL)	Maximum Value (μg/mL)	Mean Value ± SD (μg/mL)	Level of Significance
2HPA	8.2	40.8	22.7± 4.1	**	9.0	27.3	19.1 ± 6.1	*
3HPA	9.0	60.8	32.1 ± 3.3	***	39.3	46.6	41.5 ± 2.7	***
7HPA	<LOQ ^a^	23.1	14.7 ± 7.1 ^b^	NS	<LOQ ^c^	5.8	2.8 ± 0.9 ^b^	***
8HPA	14.2	42.6	26.8 ± 3.5	NS	7.7	44.3	22.5 ± 8.0	NS
9HPA	13.2	45.6	17.6 ± 5.2	**	7.5	18.8	12.8 ± 3.9	NS
10HPA	6.2	33.1	20.7 ± 4.2	NS	5.2	7.8	5.8 ± 1.1	***
11HPA	14.4	89.2	42.9 ± 4.7	***	6.5	18.9	13.5 ± 2.6	NS
16HPA	15.3	82.1	37.2 ± 5.1	***	17.1	40.8	25.1 ± 5.2	**
2HSA	10.0	51.8	23.5 ± 2.6	**	5.8	32.6	20.1 ± 6.8	NS
3HSA	12.1	54.4	33.8± 6.6	***	12.9	38.3	25.3 ± 6.3	***
7HSA	38.3	378.8	175.1 ± 3.4	***	21.5	48.0	38.2 ± 8.2	**
8HSA	<LOQ	<LOQ	0.5 ± 0.0 ^b^	-	<LOQ	<LOQ	0.5 ± 0.0 ^b^	–
9HSA	8.9	43.4	20.1 ± 4.7	***	6.4	10.8	8.6 ± 1.5	**
10HSA	31.2	250.1	72.4 ± 6.1	***	18.0	75.4	39.1 ± 7.2	**
12HSA	<LOQ ^a^	22.3	17.9 ± 5.3 ^b^	NS	5.0	11.1	9.1 ± 2.1	**
3HCA	<LOQ ^d^	18.9	7.7 ± 4.1 ^b^	***	11.8	25.2	18.8 ± 3.8	***
3HLA	<LOQ ^a^	36.8	13.2 ± 3.8 ^b^	*	6.0	15.2	11.4 ± 2.3	NS
3HMA	<LOD ^d^	19.5	12.5 ± 4.3 ^b^	**	6.1	20.8	16.1 ± 3.5	NS
3HPDA	<LOQ ^e^	18.1	4.5 ± 3.1 ^b^	***	8.5	15.6	11.5 ± 1.9	NS

Content lower than LOQ in ^a^ 7, ^c^ 10, ^d^ 8, ^e^ 13 samples; ^b^: the mean value was determined using medium-bound approach; SD: standard deviation; NS: *p*> 0.05. * *p* < 0.05, ** *p* < 0.01, *** *p* < 0.001.

## Supplementary Materials

The following are available online at https://www.mdpi.com/1420-3049/25/17/3947/s1: Figure S1: MS/MS spectra of 2HPA (A), 3HPA (B), 7HPA (C), 8HPA (D), 9HPA (E), 10HPA (F), 11HPA (G), 16HPA (H), 2HSA (I), 3HSA (J), 7HSA (K), 8HSA (L), 9HSA (M), 10HSA (N), 12HSA (O), 3HCA (P), 3HLA (Q), 3HMA (R) and 3HPDA (S), Figure S2: Principal component analysis (PCA) Scree plot of HFAs from cow and goat milk.
