# Supplementary material for: A Liquid Chromatography-High Resolution Mass Spectrometry (LC-HRMS) Method for the Determination of Free Hydroxy Fatty Acids in Cow and Goat Milk
Source: Molecules. 2020 Aug 29;25(17):3947. doi: 10.3390/molecules25173947 (PMC7504762; doi:10.3390/molecules25173947)
Supplement: Supplementary file 1 [file molecules-25-03947-s001.pdf]

SUPPLEMENTARY MATERIALS

A Liquid Chromatography-High Resolution Mass Spectrometry (LC-HRMS) Method for the Determination of Free Hydroxy Fatty Acids in Cow and Goat Milk

Maroula G. Kokotou, Christiana Mantzourani, Asimina Bourboula, Olga G. Mountanea and George Kokotos*

Department of Chemistry, National and Kapodistrian University of Athens, Athens 15771, Greece

**Table of Contents**	**Page**
**Figure S1.** MS/MS spectra of 2HPA (A), 3HPA (B), 7HPA (C), 8HPA (D), 9HPA (E), 10HPA (F), 11HPA (G), 16HPA (H), 2HSA (I), 3HSA (J), 7HSA (K), 8HSA (L), 9HSA (M), 10HSA (N), 12HSA (O), 3HCA (P), 3HLA (Q), 3HMA (R) and 3HPDA (S).	**S2**
**Figure S2.** Principal component analysis (PCA) Scree plot of HFAs from cow and goat milk**.**	**S5**

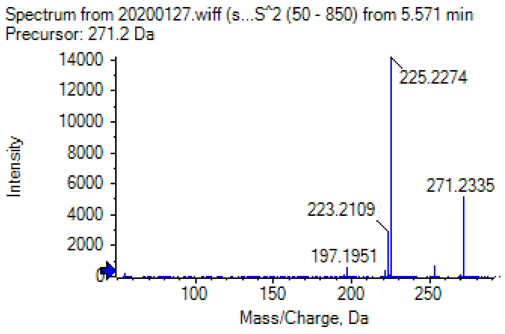
Spectrum from 20200127.wiff (s...S^2 (50 - 850) from 5.571 min
Precursor: 271.2 Da
225.2274
271.2335
223.2109
197.1951
Intensity
Mass/Charge, Da
(A)

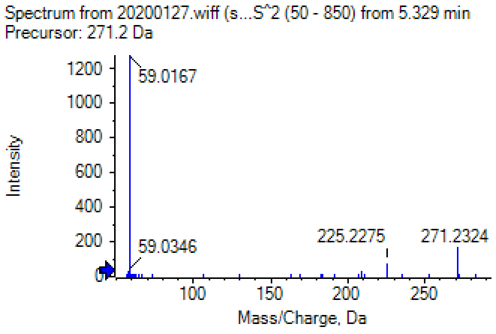
Spectrum from 20200127.wiff (s...S^2 (50 - 850) from 5.329 min
Precursor: 271.2 Da
59.0167
59.0346
225.2275
271.2324
Intensity
Mass/Charge, Da
(B)

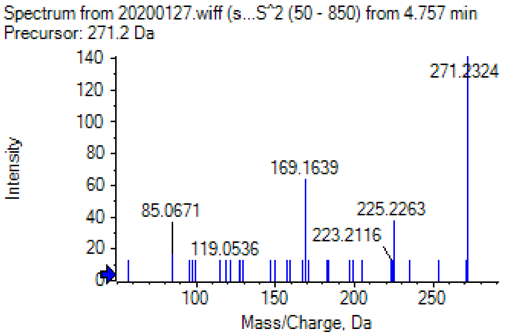
Spectrum from 20200127.wiff (s...S^2 (50 - 850) from 4.757 min
Precursor: 271.2 Da
271.2324
169.1639
85.0671
225.2263
223.2116
119.0536
Intensity
Mass/Charge, Da
(C)

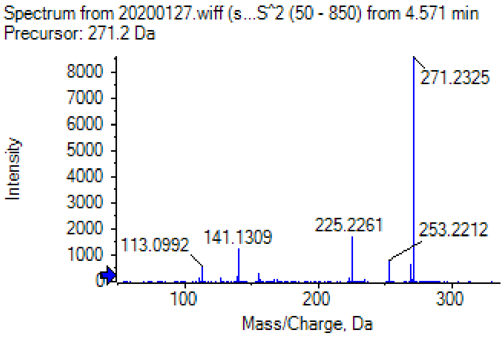
Spectrum from 20200127.wiff (s...S^2 (50 - 850) from 4.571 min
Precursor: 271.2 Da
271.2325
225.2261
253.2212
113.0992
141.1309
Intensity
Mass/Charge, Da
(D)

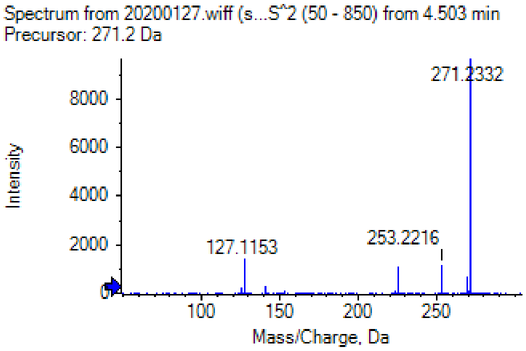
Spectrum from 20200127.wiff (s...S^2 (50 - 850) from 4.503 min
Precursor: 271.2 Da
271.2332
253.2216
127.1153
Intensity
Mass/Charge, Da
(E)

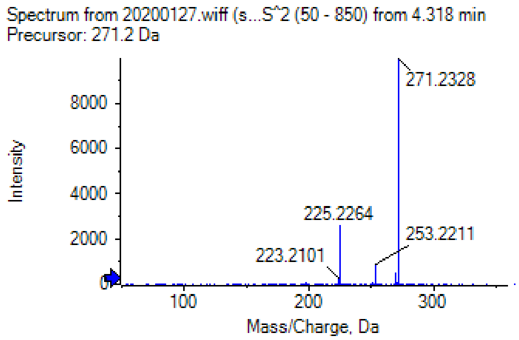
Spectrum from 20200127.wiff (s...S^2 (50 - 850) from 4.318 min
Precursor: 271.2 Da
271.2328
225.2264
253.2211
223.2101
Intensity
Mass/Charge, Da
(F)

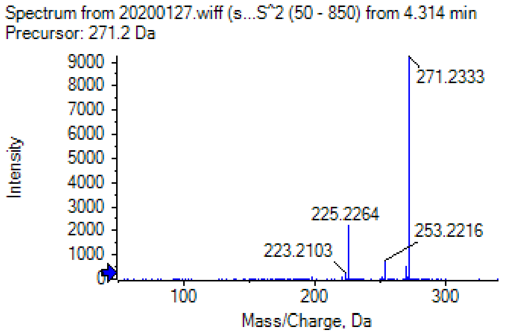
Spectrum from 20200127.wiff (s...S^2 (50 - 850) from 4.314 min
Precursor: 271.2 Da
271.2333
225.2264
253.2216
223.2103
Intensity
Mass/Charge, Da
(G)

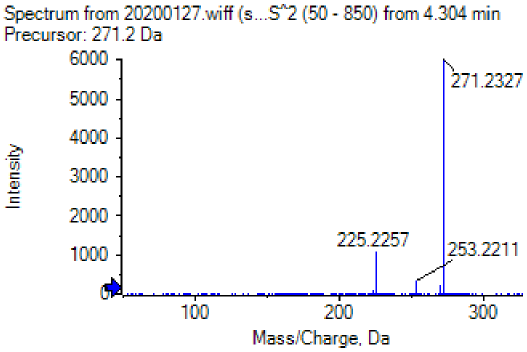
Spectrum from 20200127.wiff (s...S^2 (50 - 850) from 4.304 min
Precursor: 271.2 Da
271.2327
225.2257
253.2211
Intensity
Mass/Charge, Da
(H)

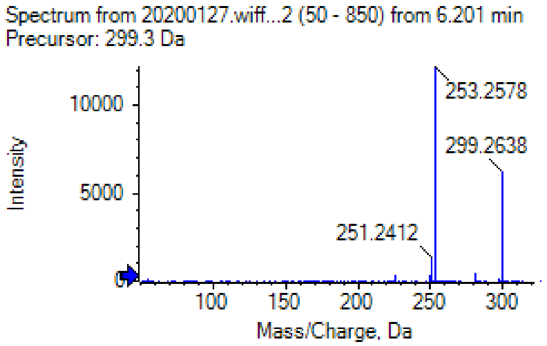
Spectrum from 20200127.wiff...2 (50 - 850) from 6.201 min
Precursor: 299.3 Da
253.2578
299.2638
251.2412
Intensity
Mass/Charge, Da

(I)

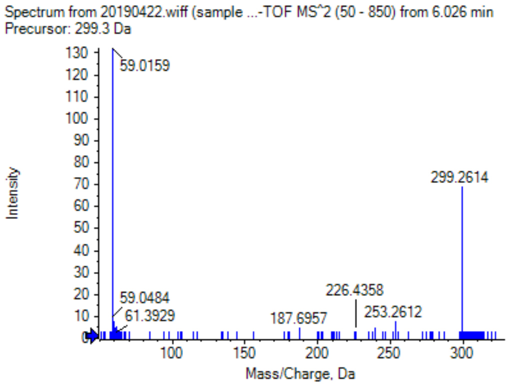
Spectrum from 20190422.wiff (sample ...-TOF MS^2 (50 - 850) from 6.026 min
Precursor: 299.3 Da
59.0159
299.2614
59.0484
61.3929
187.6957
226.4358
253.2612
Intensity
Mass/Charge, Da

(J)

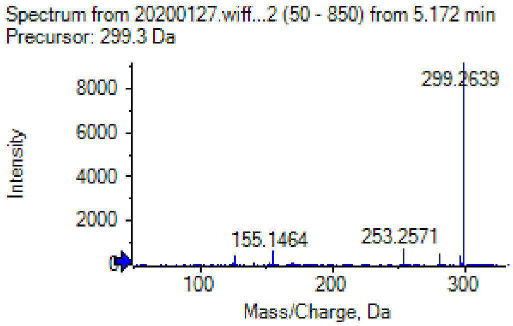
Spectrum from 20200127.wiff...2 (50 - 850) from 5.172 min
Precursor: 299.3 Da
299.2639
155.1464
253.2571
Intensity
Mass/Charge, Da

(K)

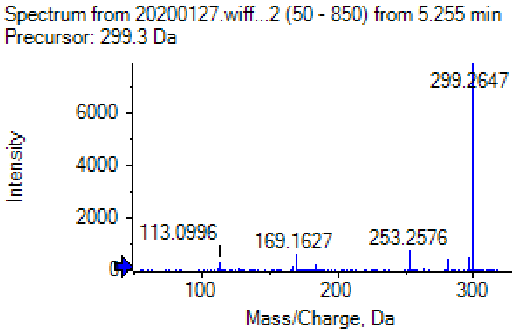
Spectrum from 20200127.wiff...2 (50 - 850) from 5.255 min
Precursor: 299.3 Da
299.2647
113.0996
169.1627
253.2576
Intensity
Mass/Charge, Da

(L)

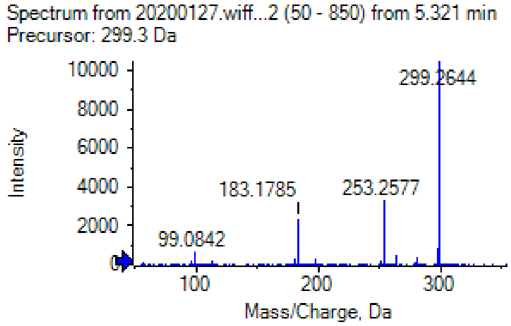
Spectrum from 20200127.wiff...2 (50 - 850) from 5.321 min
Precursor: 299.3 Da
299.2644
183.1785
253.2577
99.0842
Intensity
Mass/Charge, Da

(M)

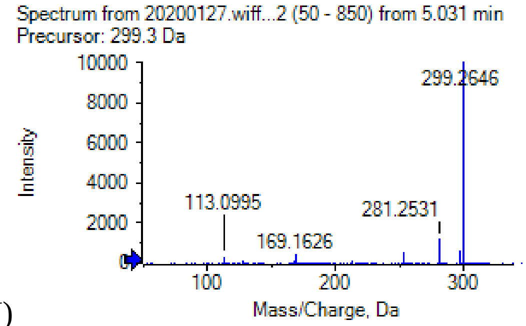
Spectrum from 20200127.wiff...2 (50 - 850) from 5.031 min
Precursor: 299.3 Da
299.2646
113.0995
281.2531
169.1626
Intensity
Mass/Charge, Da

(N)

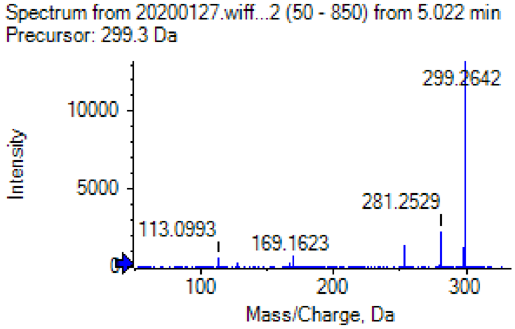
Spectrum from 20200127.wiff...2 (50 - 850) from 5.022 min
Precursor: 299.3 Da
299.2642
281.2529
113.0993
169.1623
Intensity
Mass/Charge, Da

(O)

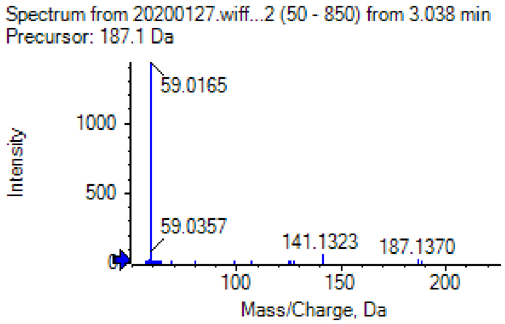
Spectrum from 20200127.wiff...2 (50 - 850) from 3.038 min
Precursor: 187.1 Da
59.0165
59.0357
141.1323
187.1370
Intensity
Mass/Charge, Da

(P)

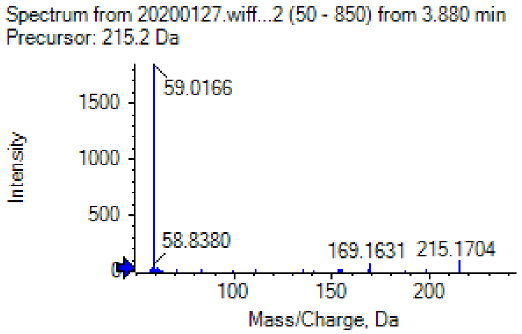

(Q)

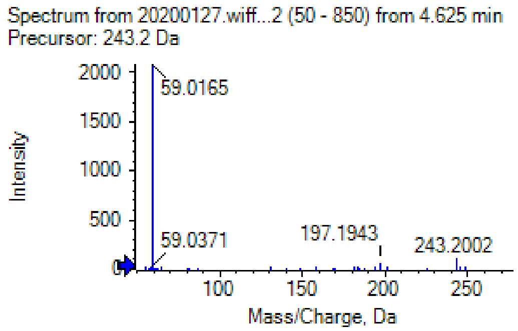

(R)

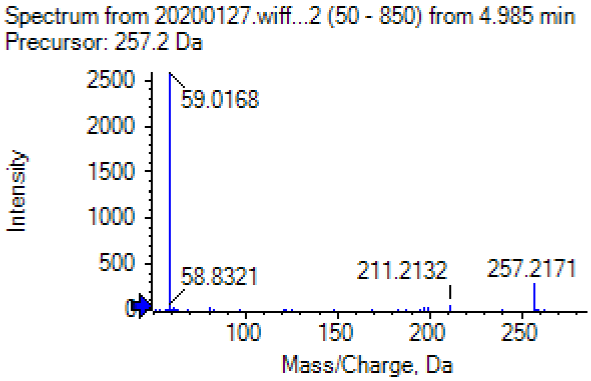

(S)

Figure S1. MS/MS spectra of 2HPA (A), 3HPA (B), 7HPA (C), 8HPA (D), 9HPA (E), 10HPA (F), 11HPA (G), 16HPA (H), 2HSA (I), 3HSA (J), 7HSA (K), 8HSA (L), 9HSA (M), 10HSA (N), 12HSA (O), 3HCA (P), 3HLA (Q), 3HMA (R) and 3HPDA (S).

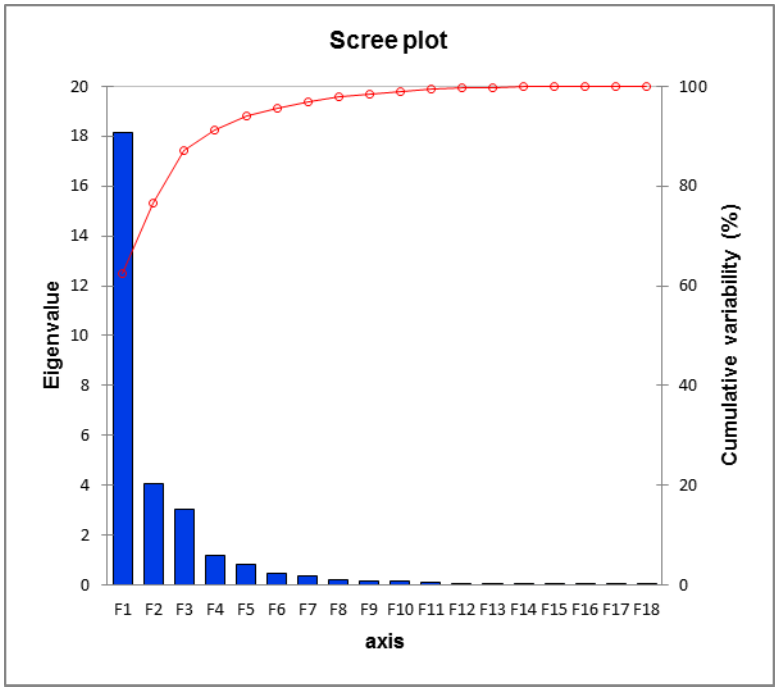

Figure S2. Principal component analysis (PCA) Scree plot of HFAs from cow and goat milk**.**
